# Editorial: Treatment and prognostic assessment of liver cirrhosis and its complications, volume II

**DOI:** 10.3389/fmed.2025.1601785

**Published:** 2025-04-29

**Authors:** Wei Zhang, Yufu Tang, Hao Wu, Andrea Mancuso, Thierry Thévenot, Xingshun Qi

**Affiliations:** ^1^Liver Cirrhosis Study Group, Department of Gastroenterology, General Hospital of Northern Theater Command, Shenyang, China; ^2^Department of Hepatobiliary Surgery, General Hospital of Northern Theater Command, Shenyang, China; ^3^Department of Gastroenterology, West China Hospital, Sichuan University, Chengdu, China; ^4^Dipartimento di Medicina Interna, ARNAS Ospedali Civico Di Cristina Benfratelli, Palermo, Italy; ^5^Department of Hepatology, University Hospital of Besançon, Besançon, France

**Keywords:** liver cirrhosis, treatment, assessment, complications, portal hypertension, variceal bleeding

From an initial discussion with the in-house editors of Frontiers on November 23, 2023, a Research Topic entitled “*Treatment and prognostic assessment of liver cirrhosis and its complications, volume II*” was launched in Frontiers in Medicine on December 12, 2023. We received a large number of submissions until the deadline on August 4, 2024. Overall, a total of 21 papers involving 157 authors were published after internal editorial assessment, external peer review, and editorial decision processes. Interestingly, at the time of writing this editorial on March 27, 2025, they have been viewed or downloaded over 10 thousand times. Herein, their contents have been briefly summarized in the following sections.

## Symptoms in liver cirrhosis

Philips from the Rajagiri Hospital, India, comprehensively reviewed several common symptoms, including malaise, fatigue, lethargy, appetite disorders, restrictive diet, malnutrition, non-cholestatic pruritus, muscle cramps, sleep disorders, mental health disorders, gastrointestinal symptoms, sexual dysfunction, pain, peripheral neurological symptoms, hair loss, and skin changes, in patients with liver cirrhosis, and summarized their management. Xie et al. from the Hebei Clinical Research Center for Digestive Diseases, China, performed a meta-analysis of 16 observational studies and demonstrated that the overall estimated prevalence of frailty was 27% in cirrhosis patients and that its occurrence was positively associated with patients who were male, older, had a lower BMI, or had worse liver function.

## Pathogenesis of liver fibrosis

There are three papers regarding the pathogenesis of liver fibrosis. Hu et al. from the Shengjing Hospital of China Medical University, China, reviewed the role of Chitinase-3-like protein 1 in the pathogenesis and diagnosis of liver fibrosis. Shamsan et al. from the Qinghai University, China, comprehensively reviewed the role of the PI3k/AKT signaling pathway in attenuating liver fibrosis. Guo et al. from the Shandong University, China, performed a Mendelian randomization study to confirm the causal effects of cigarette smoking on liver fibrosis and cirrhosis.

## Assessment of liver cancer

There are three papers regarding the assessment of risk factors and prognostic factors for liver cancer. Dong et al. from the General Hospital of Northern Theater Command, China, retrospectively analyzed the association of ABO blood groups and rhesus factor with primary liver cancer in liver cirrhosis. The researchers did not find any significant association between them in patients with cirrhosis. Qiao et al. from the Beijing You'an Hospital of Capital Medical University, China, developed a nomogram by combining gamma-glutamyl transpeptidase-to-platelet ratio, age, and hemoglobin to predict the overall survival of patients with compensated cirrhosis and hepatocellular carcinoma who were treated with local ablation. They demonstrated good predictive performance of this nomogram in such patients. Luo et al. from the Shulan (Hangzhou) Hospital Affiliated to Zhejiang Shuren University Shulan International Medical College, China, evaluated the role of γ-glutamyl transferase to serum albumin ratio in assessing the survival of patients with hepatocellular carcinoma who underwent liver transplantation. They demonstrated that the γ-glutamyl transferase to serum albumin ratio ≥2.04 was independently associated with recurrence-free and overall survival.

## Assessment of portal hypertension

There are five papers regarding the assessment of portal hypertension. Shanka et al. from the I.M. Sechenov First Moscow State Medical University, Russia, systematically reviewed the evidence regarding non-invasive methods for diagnosis of portal hypertension in liver cirrhosis secondary to non-alcoholic fatty liver diseases or metabolic dysfunction-associated steatotic liver diseases. They suggested that the measurement of liver and spleen stiffness offered good diagnostic evaluation of clinically significant and severe portal hypertension. Mao et al. from the First Affiliated Hospital of Anhui Medical University, China, explored the diagnostic performance of esophageal varices' diameters, which were measured using a virtual ruler under endoscopy, to assess portal pressure gradient, which in turn was measured using interventional radiology. Cao et al. from the same study group also performed a multicenter study to evaluate the diagnostic performance of esophageal varices diameter, which was also measured using a virtual ruler under endoscopy, to predict the risk of early rebleeding within 6 weeks after endoscopic variceal ligation. The novelty of the two studies is in the use of a virtual ruler-based measurement of esophageal varices. The researchers found that an esophageal varices diameter of >1.1 cm and ≥1.4 cm with a virtual ruler were related to a significantly increased portal pressure gradient level of 20 mmHg and early rebleeding in cirrhosis, respectively. Li et al. from the Women and Children's Hospital of Chongqing Medical University, China, developed and internally validated a clinical-radiomics nomogram by combining prothrombin time, sarcopenia, and radiomics score to predict the occurrence of upper gastrointestinal bleeding in liver cirrhosis. Notably, the radiomics score was established by extracting 11 different features on CT images. Ye et al. from the West China Hospital of Sichuan University, China, evaluated the association of metabolites with severe portal hypertension indicated by a hepatic venous pressure gradient of >16 mmHg in Tibetan patients with liver cirrhosis. Notably, by using metabolomics, the researchers identified pisumionoside and N-decanoylglycine as promising biomarkers for severe portal hypertension.

## Assessment of prognosis from other aspects

Gülcicegi et al. from the University Hospital Cologne, Germany, evaluated the role of dynamic changes in spleen stiffness after starting acute decompensation treatment in hospitalized cirrhotic patients. They found a gradual decrease in spleen stiffness after effective treatment. Que et al. from the Daping Hospital of the Army Medical University, China, developed a novel prognostic model by combining age, ascites, albumin, prothrombin time, total bilirubin, and sodium in patients with viral hepatitis-related cirrhosis who were treated by transjugular intrahepatic portosystemic shunt (TIPS). They found that the novel post-TIPS prognostic model had good predictive performance. Wang S. et al. from the Peking University People's Hospital, China, retrospectively evaluated the risk factors of the first liver-related readmission within 30–90 days after the index hospitalization. They found that hepatic encephalopathy, ascites, and spontaneous bacterial peritonitis caused a higher risk of prehospitalization.

## Treatment of liver cirrhosis-related complications

There are three case reports, one retrospective study, and one meta-analysis regarding the treatment of liver cirrhosis-related complications. Liu et al. from the Beijing Xiaotangshan Hospital, China, reported a case of hepatic myelopathy that was successfully treated by liver transplantation and comprehensive rehabilitation training. Sun et al. from the Gansu Provincial Hospital, China, reported a case of gastric variceal bleeding that was successfully treated by TIPS via the mesenteric venous pathway. Wang R. et al. from the General Hospital of Northern Theater Command, China, reported a case of liver cirrhosis with acute symptomatic portal vein system thrombosis that developed after endoscopic variceal therapy and was successfully treated by immediate anticoagulation. Tie et al. from the Xijing Hospital of Digestive Diseases of the Air Force Medical University, China, evaluated the efficacy of variceal embolization for primary prophylaxis of variceal bleeding in cirrhotic patients who were not suitable candidates for non-selective beta blockers or endoscopic treatments. They demonstrated that variceal embolization achieved a high rate of technical success and low rates of recurrence and severe complications. Yang et al. from the People's Hospital of Guangxi Zhuang Autonomous Region and Guangxi Academy of Medical Sciences, China, conducted a meta-analysis and showed the benefits of probiotics for hepatic encephalopathy reversal, liver function, quality of life improvement, and gut dysbiosis regulation in patients with cirrhosis.

We hope that the findings from the papers published in this Research Topic can have a substantial impact on real-world clinical practice. We also expect to initiate Volume III of the Research Topic in the near future and receive more interesting and valuable papers.

